# Blockchain-enabled quality by design system for clinical trials

**DOI:** 10.3389/fmed.2025.1546897

**Published:** 2025-12-04

**Authors:** Reza Vatankhah Barenji, Reza Ebrahimi Hariry

**Affiliations:** 1 Department of Engineering, School of Science and Technology, Nottingham Trent University, Nottingham, United Kingdom; 2 School of Life Sciences, University of Nottingham, Nottingham, United Kingdom

**Keywords:** clinical trials, quality by design, blockchain technology, drug development, PDCA

## Abstract

**Introduction:**

Blockchain technology offers a secure and distributed approach to data management that can strengthen the Quality by Design (QbD) framework in clinical trials. Integrating blockchain with QbD can enhance data integrity and promote participant safety.

**Methods:**

A blockchain-enabled QbD architecture was developed to support systematic quality improvement in clinical trials. The interactions among its components and peers were described, and a prototype was implemented using Hyperledger Fabric. Data from a pilot clinical trial were used to evaluate its applicability.

**Results:**

The prototype demonstrated that the proposed architecture efficiently facilitates immutable data exchange, enhances traceability among QbD activities, and supports secure collaboration between stakeholders. The system improved data consistency and enabled automated verification of trial processes.

**Discussion:**

This study shows that blockchain technology can effectively enhance QbD implementation in clinical trials by improving data integration, transparency, and safety monitoring. The proposed architecture provides a feasible and scalable model for future clinical data management systems.

## 1 Introduction

The estimations reported that the costs of developing a new drug exceed 1.2 billion dollars and take an average of 12 years from creation to market, causing pharmaceutical companies to experience a pronounced “profitably gap” (1). As pressures on the pharmaceutical industry continue to mount, quality and safety issues become a greater concern. Companies should build safety, quality, and efficacy into their new pharmaceutical products as early as possible (2). Participant safety in clinical trials (CTs) is the pivotal aspect of quality in the drug development process, and all stakeholders attempt to collaborate proactively to ensure the safety of participants. The evaluation and management of the risks and minimization strategies are fundamental requirements for regulating authorities (3). Data integrity in CTs is the next complementary piece of quality to provide reliable and accurate data since missing data have seriously compromised inferences in CTs (4). Some works have promoted several approaches to quality improvement in CTs. Mainly, they focused on the monitoring process based on Good Clinical Practice (GCP) (5–7). GCP is the global quality standard to ensure the quality conduct of the CTs (8). Although this standard has been defined for design, conduct, monitor, audit, analyze, and report the CTs, serious concerns related to the structure and delivery of GCP remain. To build quality into a clinical process rather than ensuring the quality of clinical service through audits and inspections that perform in GCP, Quality by Design (QbD) has been introduced (9).

QbD is a systematic approach to development that begins with predefined objectives and emphasizes product and process understanding and process control, based on sound science and quality risk management (10). It emphasizes building quality into a process from the beginning and has been successfully utilized in drug manufacturing (11). The major enabler in implementing QbD is to extract, review, and use the knowledge coming from diverse sources and then assess quality attributes and improve conduction strategies. Therefore, effective data management is the key factor for the successful implementation of QbD (12). The implementation of QbD in the initial phases of drug development has been highly successful. However, QbD in clinical and pharmacovigilance areas is still in its infancy, mainly because its implementation requires a high level of data integrity among stakeholders and operational units. This complexity may ultimately affect participant safety (13). With the growing emphasis on enhanced process understanding for quality improvement, the need for establishing an efficient data management system for QbD is more important than ever (14).

To gain greater efficiencies and higher quality in clinical research, QbD needs to embrace newly merged technological tools and approaches to manage generated knowledge well before, during, and after the CTs’ conduction. Perhaps the most important prerequisite to innovation is employing Blockchain Technology (BCT) to leverage data management. BCT might play an important role in the successful implementation of QbD and enhance the quality of CTs by facilitating efficient transfer and utilization of data. Blockchain technology, with its unique characteristics, offers an immutable way to store data and ensures data integrity among stakeholders and units. Furthermore, the use of smart contracts can automate processes to some extent, thereby enhancing participant safety and minimizing the risk of harm (15).

This study provides a blockchain-enabled QbD architecture in the clinical trial stage of drug development aiming to improve the quality of CTs by first highlighting the factors that affect the safety of the participants, defining measurement approaches and safe domains/states for the selected factors; second, conduct the trials, and achieve the amounts of the factors for the participants; third, performing root-cause analyses; and finally, develop safety control strategies. The architecture improves the quality of the CTs by enhancing patient safety through the use of immutable safety-related data on the blockchain and boosting data integration among the QbD activities. A case study simulation is used as a “proof of concept.”

## 2 Literature review

In the next subsections, the building block of BCT is presented, and some applications of this technology in clinical research are reviewed; later, the QbD approach is stated, and the importance of data in QbD is highlighted.

### 2.1 Blockchain technology and its applications in clinical research

BCT is a huge, public, secure, and decentralized data store of ordered records. It is a distributed ledger (database), structured as a chain of blocks that each holds transactions. The ledger is owned by no one and is controlled by the nodes of the distributed network, but not by any centralized authority or trusted third party. The data of the ledger is immutable and auditable and cannot be hacked, erased, or stolen since a copy of the ledger is stored over the nodes (users) of the decentralized network, authentications are verified through cryptographic techniques, and all the transactions are timestamped and accomplished through the use of cryptographic hashing (16). By the hashing process, data with variable length is altered into a fixed-length digest, which is called a Hash. If any change in the input data happens, the hash will be changed unpredictably. Any block in the ledger holds the hash of the previous block and the transactions that are used to generate the block’s hash. In case a change happened in any of the previous blocks (the hash of the block will be changed), the subsequent hash would no longer be valid. A new transaction can be added to the block if the majority of nodes reach consensus. Trust is provided by the community of the nodes through encoding the smart contracts (17).

There are mainly four types of BCT ledgers, namely, public, private, consortium, and hybrid. The former is an open-source ledger that allows anyone to participate in the network, a private ledger restricts who is allowed to enter the network, in consortium ledger allows more than one node can manage the entrances in the network, and the latter is a combination of private and public ledger (18).

The integrity of data in a CT is essential, but the current data management process is too complex and highly labor-intensive (19, 20). Massive volume and variety of data generated during multiple phases and multi-year studies in CTs bring several challenges, such as privacy issues, costs, results in reproducibility, data integrity and sharing, patient enrolment and recruitment, and protocol compliance (21–23). To tackle these obstacles, pharma and biotechnology companies are starting to move from currently employed clinical data management systems (e.g., Oracle Clinical software, SigmaSoft’s DMSYS Software) toward new decentralized platforms (24, 25). BCT has the potential to address some of these challenges. Hirano, T. et al. (2020) developed and tested a BCT-based data management system for clinical use in breast cancer to validate a system that enables the security of medical data in a CT using blockchain technology (26). Gordon, W. J. et al. (2018) looked at how BCT might facilitate a transition to Patient-Driven Interoperability through five mechanisms: (1) digital access rules, (2) data aggregation, (3) data liquidity, (4) patient identity, and (5) data immutability (27).

This technology is applied by Santos, J. A. et al. (2021) as a mechanism to protect Electronic Health Records (EHRs), patient health data, and mobile health (m-Health) (28). Cheng et al. (2020) proposed a network model of a Medical Cyber-Physical System (MCPS) based on blockchain to design a secure medical data sharing scheme (29). BCT may overcome many issues of medical data (EHR and EMR), such as security challenges (30), data storing, managing, and protection (31), integration and access controlling (32), and privacy of users in the eHealth world (33). This technology is a safe platform for storing sensitive information, including electronic healthcare records, clinician notes, e-prescribing, analysis results, and protocols. It might enhance drug development research by empowering the research community with a secure network for sharing data (34). The convergence of BCT and CTs is still in its infancy, and very limited literature is available on this subject. At a high level, BCT and smart contracts can be used in patient recruiting and enforcement of human subject regulations to manage and monitor data in multi-site CTs, a framework (35, 36). A system named “BlockTrial” has been developed to facilitate users in executing trial-related smart contracts on a private network (37). A BCT-based solution is introduced to manage consent information between patients and stakeholders (38). To deal with the dynamic nature of consent management, a blockchain-enabled framework has been presented (39).

More recently, there has been growing attention on the use of BCT in clinical research due to its potential to enhance data integrity, transparency, and security. Leiva and Castro (2025) highlight BCT’s potential to improve data integrity, transparency, and security in clinical research, particularly when integrated with AI to enhance patient recruitment, data analysis, and trial management in low-resource settings (40). Omidian (2024) also underscores the advantages of AI-BCT platforms, including improved data sharing, privacy, and decision-making, while noting challenges like scalability, latency, and regulatory compliance (41). Kasahara et al. (2024), through a systematic review, examine digital tools—e.g., social media, e-consent, ML, and blockchain—for improving trial enrollment, especially among minorities (42). Despite their promise, consistent evidence of increased diversity remains lacking. Castro et al. (2024) conduct a bibliometric analysis of 107 studies, identifying seven thematic areas where BCT enhances data management, consent, and transparency (43). Moatari-Kazerouni et al. (2024) propose a BCT framework addressing stakeholder communication, data validity, and regulatory compliance, streamlining collaboration and reducing trial inefficiencies (44). Cihan et al. (2025) use 34 studies to develop a conceptual model guiding BCT platform design, focusing on immutability, privacy, and interoperability, while addressing legal and technical challenges (45). In a practical application, Cihan (2025) implements a Hyperledger Fabric system for COVID-19 reporting, integrating FAIR principles and smart contracts to enhance data transparency, automation, and usability (46). Sethi et al. (2025) emphasize BCT’s role in preventing tampering, ensuring regulatory oversight, and maintaining confidentiality across multi-center trials (47). Nnadiekwe et al. (2024) developed a patient-centric Ethereum-based system with smart contracts to manage consent and adverse event reporting, promoting trust and real-time data sharing (48). Mahdavi (2025) and Pillai (2024) both review the complementary strengths of BCT and AI in enhancing data security, analytics, and regulatory efficiency, grounding their claims in case studies and real-world frameworks (49, 50). Mackey et al. (2025) address youth underrepresentation in trials using co-designed digital tools that outperform traditional outreach in boosting engagement, stressing user involvement in tool development (51). Byiringiro (2025) and King (2024) both critique the lack of diversity in clinical trials, proposing inclusive strategies rooted in structural reform and social determinants frameworks (52, 53). Peiper (2024) links clinical trial participation to awareness of ClinicalTrials.gov and digital health engagement, suggesting digital tools as effective levers for increasing trial involvement (54).

### 2.2 Quality by design

The QbD approach is an approach aiming to guarantee the quality of designing, developing, and manufacturing processes of medicines by employing statistical, analytical, and risk-management methodology (55). According to the ICH Q8 guideline, it is “a systematic approach to development that begins with predefined objectives and emphasizes product and process understanding and process control, based on sound science and quality risk management” (56). QbD has been performed in many aspects of biotechnological and pharmaceutical developments, including, but not limited, biopharmaceuticals (57), bioprocessing (58), abbreviated new drug applications (ANDAs) (59), drug formulation and delivery (60–62), pharmaceutical set-up (63), analytical methods (64–66), biotechnological products (67), drug development (68), nano-pharmaceutical products (69, 70), monoclonal antibodies (71), and CTs (9, 72, 73).

Pharmaceutical QbD includes designing production processes to guarantee predefined formulation quality and enhance development capability, speed, and formulation design (74). Quality in QbD should be designed into a product, which was supported by Guidance for Industry PAT-a framework for innovative pharmaceutical development, manufacturing, and quality assurance, with “quality cannot be tested into products; it should be built-in or should be by design” expression (75). The progression of QbD principles in the pharmaceutical industry can be monitored with the guidelines based on regulatory processes (ICH PAT Guideline, 2004) (76), ICH Q8 (R2) (Pharmaceutical Development, 2014) (56), ICH Q9 (Quality Risk Management, 2014) (77), ICH Q10 (Pharmaceutical Quality System, 2009) (78), ICH Q11 (Development and Manufacture of Drug Substance, 2011) (79), and ICH Q12 (technical and regulatory considerations for pharmaceutical product lifecycle management, 2020) (80). The main objectives of QbD are to obtain predefined quality specifications based on clinical performance, reduce formulation variability, and develop a post-approval change organization by improving product and process design, understanding, and control to prevent product variability that causes rejects, scrap, and re-processing (81).

For decades, QbD has played a vital role in drug design and manufacturing; however, it has just begun to take a role in CTs. The QbD-based system design should take into account regulatory frameworks such as ICH M11, which provides technical specifications for the structure and content of clinical trial protocols (82). Recently, QbD has been used in some clinical research and trials for improving the safety, quality, efficacy, cost, and time to market a potential new drug. In CTs, principally, quality is the sponsor’s judgment about the overall credibility of the results. The common approaches for quality improvement are focused on the monitoring processes based on GCP (56), and according to the ICH E8 (R1) Guideline, QbD in clinical research proactively ensures that the quality of a study and the reliability of the results will be improved by considering participants’ safety and risk management approaches (83).

Plan-Do-Check-Act (PDCA) is a framework upon which the QbD in CTs is based (84). Plan: (a) identify the factors that are critical to quality and must be met during the conduct of the CTs, (b) determine the procedures that will enable quality measurement for the predefined quality factors, and (c) systematically identify the safe domains of factors of the planned CT, aiming to identify and prioritize risks to quality. Do: implement quality risk management plans during and after the conduct of the CTs. Check: measure/monitor quality performance to assess whether quality factors are being met to enable identification of the risks, their levels, and roots. Act: respond to quality issues with appropriate corrective and/or preventive control strategies.

The flow and integration of data among the PDCA in QbD play a vital role in the producibility of the approach and boosting the quality (85). Real-time data exchange among the activities in an integrated manner can be realized through incorporating in PDCA architecture. It plays a key role in facilitating efficient transfer and utilization of information among the activities, and it facilitates reviewing the information coming from a disparate variety of sources and then using it to assess the criticality of the various quality attributes. QbD enabled with a well-structured data management system might be effective in managing the flow of information toward the development of process strategies, and technology transfer, as well as using this information towards continuous improvement of the processes (13). A commonly centralized data sharing system is used for exchanging and sharing data among the activities and the involved enablers of QbD, which is not quite effective (63). These kinds of repository systems may not be operational since the enablers are geographically distributed and heterogeneous in terms of the operating system. A decartelized data management is required to level up the QbD in CTs. A distributed unauthorized data repository and management system may improve the data management process, which universally facilitates data sharing. In addition, the generated data by QbD is personal sensitive data, and their misuse might harm the agreements, corporation policies, and participant privacy. The data of the QbD should be stored in a high level of security and should not be manipulable by any authority. It is not surprising that, well, data integration among the QbD activities and enablers will improve the quality. In this study, BCT is embedded in the QbD approach, aiming to develop a distributed data sharing and management system to overcome the above-mentioned shortcomings.

BCT can effectively address several weaknesses in QbD knowledge management. One major limitation of traditional QbD systems is the fragmentation of data across different departments and organizations, which often leads to inconsistency and reduced data reliability (86). BCT overcomes this by providing a shared, tamper-proof ledger that enables real-time data visibility and synchronization among all authorized stakeholders (87). Another challenge in QbD is maintaining data integrity and traceability. BCT ensures that all records are time-stamped, cryptographically secured, and immutable, creating a transparent and verifiable audit trail for every design or process change. Collaboration across multiple stakeholders is also often hindered by version control issues and information silos. By establishing a single, consensus-driven source of truth, BCT enables all participants to access the same verified version of data, improving communication and decision-making (88). Furthermore, QbD knowledge is often underutilized because valuable insights from one phase of development are not easily transferred to others. Through its decentralized and permanent data storage, BCT supports secure knowledge sharing and reuse while protecting confidentiality through permissioned access. Smart contracts can automate compliance checks, process validation, and documentation tasks, reducing manual errors and ensuring adherence to regulatory standards (89). Collectively, these features strengthen the integrity, transparency, and efficiency of QbD knowledge management and contribute to more reliable and safer clinical and pharmaceutical development processes.

The adoption of the PDCA framework in clinical trials offers a structured, iterative approach that aligns closely with the QbD paradigm, fostering continuous quality improvement across all trial phases. In the Plan phase, critical trial components—including objectives, eligibility criteria, risk assessments, and safety endpoints—are systematically defined. BCT can enhance this phase by providing immutable documentation and version control of protocol elements. For instance, in designing a bioequivalence trial, a safety concern such as QT interval prolongation can be formally linked to pre-set safety thresholds and recorded on the blockchain to support traceability and regulatory compliance. In the Do phase, clinical execution can adhere to predefined parameters, with BCT ensuring data integrity and authenticity through cryptographic hashing and consensus mechanisms. All operational data—such as enrollment logs, lab results, and adverse event reports—can be uniformly committed across sites to a distributed ledger, enabling real-time validation and auditability.

In the Check phase, ongoing monitoring and interim analyses can assess compliance with protocol specifications and quality benchmarks. BCT can facilitate centralized, tamper-evident verification of data origin, timestamps, and investigator credentials, allowing oversight bodies to evaluate safety signals or deviations promptly. For example, an emerging trend in adverse events can be reliably identified and contextualized using BCT-based datasets. Finally, the Act phase can translate these insights into protocol refinements or operational adjustments—such as revising dosing strategies or tightening inclusion criteria based on observed risks in specific subpopulations. These amendments can be recorded immutably, ensuring transparency, regulatory defensibility, and reproducibility across the trial continuum. By embedding this closed-loop quality system, the PDCA model can enhance responsiveness, data integrity, and stakeholder trust in clinical research.

## 3 Proposed blockchain embedded with quality by design architecture in clinical trials

Quality by design in CTs is a systematic approach that aims to ensure quality and satisfy the regulators by applying risk management in the entire process of clinical studies. The quality of a CT significantly depends on the protection of participants and data integration. Therefore, based on this argumentation, the main elements of QbD should be redefined as Quality Targets (QTs), Quality Attributes (QAs), Critical Safety Attributes (CSAs), and Safety Control Strategies (SCSs). QTs in clinical trials are the reliability and credibility of information collected during the clinical research process. Reliability refers to all the aspects that affect the safety of the participants. In addition, QAs are aspects that affect the reliability and credibility of results. The QAs that are directly related to participants’ safety in clinical trials are considered the CSAs. Thus, the clinical processes with high levels of risks and probabilities of occurrence and impacts under CSAs should be highlighted for risk management. As a result, suitable execution domains for the selected processes would be defined in a way that the risks would be mitigated. These appropriate execution domains for the processes are called safety control strategies. The SCSs should be considered during the trials, analysis of the risks, and impacts of the processes.

**Figure 1:**
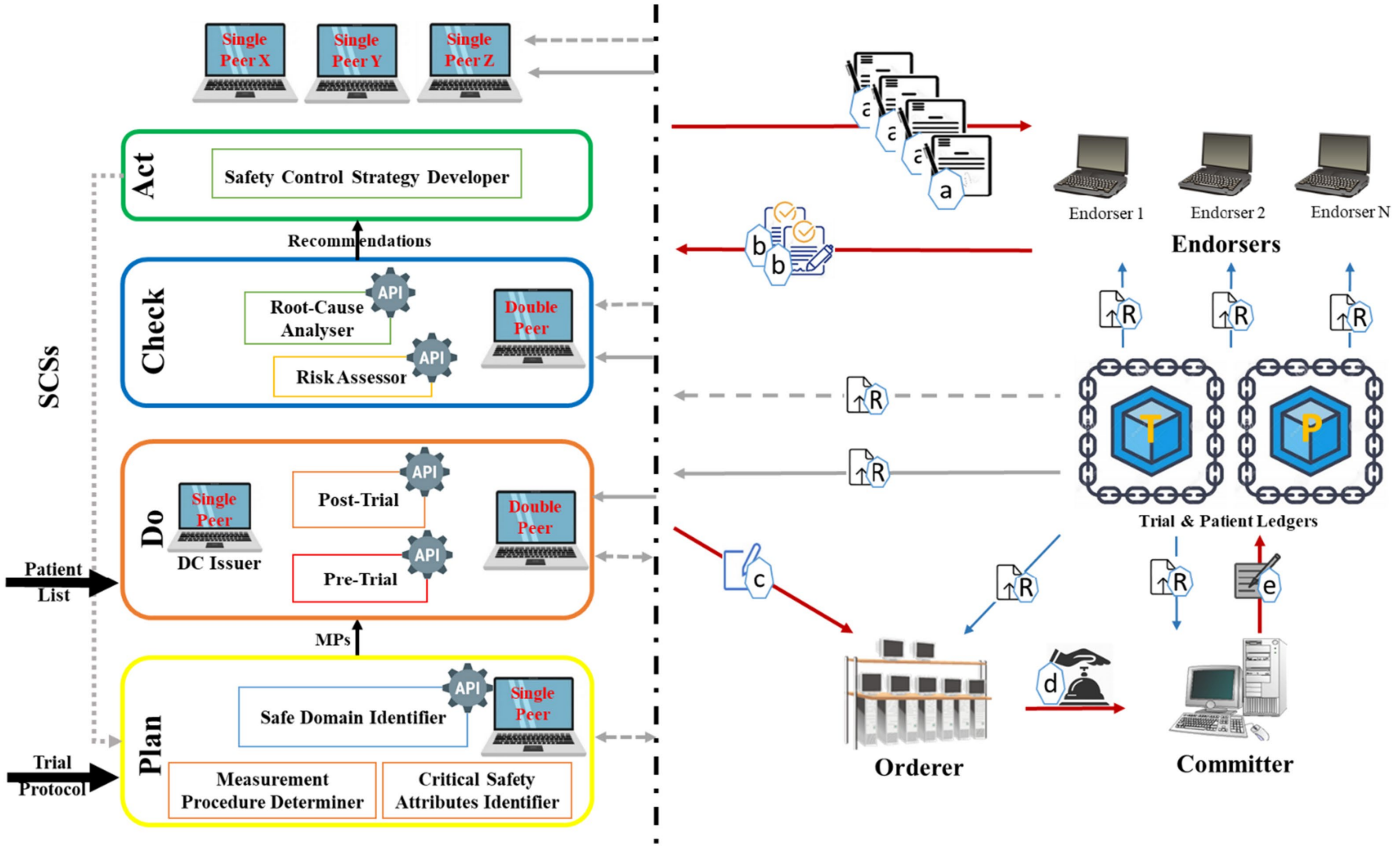
Blockchain-embedded Quality by Design (QbD) architecture for clinical trials.

Figure 1 represents the developed architecture that embeds QbD with the BCT for CTs. The QbD phases in the architecture are represented as a rectangle, and the BCT is represented by peers shown as computer symbols. In the architecture, the QbD phases are named plan, do, check, and act. Initially, in “Plan,” the Critical Safety Attribute Identifier (CSAI) unit highlights the factors that affect(s) on CSAs, the Measurement Procedure Determiner (MPD) unit provides the measurement approach of the selected factors, and the Safe Domain Identifier (SDI) unit provides the safe domains/states of the selected factors. The decisions made by these units are based on information available on the trial protocol. In the “Do,” the Pre-Trial (PrT) unit determines the amounts or states of the factors using the recommended measurement approaches. The trials will be conducted when the amounts/states of the factors provided by the PrT are in acceptable domains/states provided by the SDI. The post-Trial (PoT) unit identifies amounts/states of the factors after clinical trials are conducted according to the measurement approaches. In “Check,” the Risk Assessor (RA) unit highlights the factors with high risk, and the Root-Cause Analyzer (RCA) unit performs root-cause analyses based on the prior and later amounts/states of the factors. Finally, the “Act” Safety Control Strategy Developer (SCSD) unit generates safety control strategies that will be used in the next cycle of the plan. Peers are a fundamental element of the BCT since they host ledgers and smart contracts. Ledger securely records transactions across many peers, making the data transparent and tamper-resistant. A smart contract is a self-executing program stored on the peer that automatically carries out agreed-upon rules or actions when certain conditions are met.

Hyperledger Fabric was selected as the blockchain framework for this architecture due to its permissioned network structure, which supports role-based access control, ensuring compliance with regulatory expectations for data governance in clinical trials. Its modular consensus mechanism supports scalable performance without relying on energy-intensive proof-of-work, while private channels enable selective data visibility—an essential feature for maintaining patient confidentiality and protocol integrity. Compared to public or semi-public platforms like Ethereum, which prioritize transparency over access restriction, Hyperledger Fabric offers the structural flexibility and privacy safeguards needed for controlled clinical research environments. In Hyperledger Fabric, peers are the nodes that maintain the ledger and run smart contracts (chaincode). Among peers, endorsers simulate transactions by executing the chaincode and providing endorsements that validate the transaction’s correctness. The orderer is a separate component responsible for ordering all transactions into a consistent sequence and packaging them into blocks. Once blocks are created by the orderer, committer peers receive these blocks and update their ledger by committing the validated transactions. Thus, endorsers verify transactions, the orderer sequences them, and committers finalize the ledger state.

Two types of ledgers are considered in the system, namely, the Trial ledger (T-ledger) and Patient ledger (P-ledger). The safe domains/states of the factors are deposited in the T-ledger, and the amounts/states of the factors for each participant are stored on the P-ledger. The peers in the system would have different obligations, namely, “single,” “Double,” “DC issuer,” “endorser,” “orderer,” and “committer.” The “single” peer host T-ledger or P-ledger and the “Double” peer host both the ledgers and their smart contracts. The “DC issuer” peer issues a digital certificate for the participants, which is known as an identity certificate. “Endorser” peer simulates the transaction proposal and then validates or refuses the requested transaction. The “Orderer” accomplishes the mining process for the new blocks of transactions, and the “committer” appends the validated transactions to the channel-specific ledger.

In the system, the QbD is in connection with BCT through the peers. These interconnections realize data exchange, sharing, and management among the phases and the entire system. The type of peer employed (‘single’ or ‘double’) depends on the kind of data that is generated or required within each phase of the QbD process. These peers realize data and/or repository from/to the ledgers. Commonly, the “endorser” peers might belong to independent third parties, and the “orderer” and “committer” peers might be authorized by a national or international health authority. Any other involved organization (i.e., regulators, third parties) that should have access to the BCT holds a “single” or “Double” peer.

### 3.1 The QbD phases, activities, and their interactions with BCT

**Figure 2:**
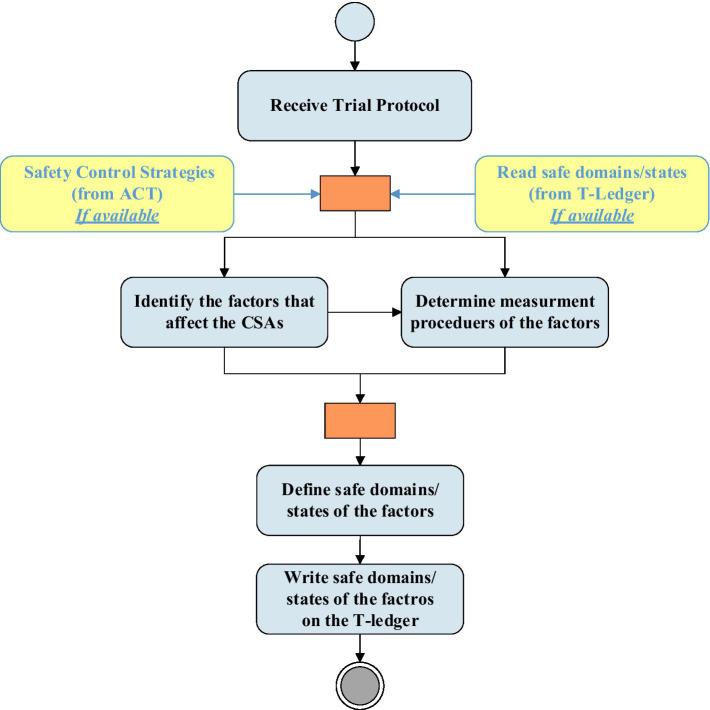
“Plan” phase activities and interactions with the BCT.

The BCT-embedded QbD is an iterative four-phase quality improvement method. Figure 2 represents the activities of the planning phase in the architecture and its interconnection with the BCT. As shown in the figure, initially, the CSAI unit screens the trial protocol of the project received from the sponsor and identifies the factors that affect critical safety attributes. The MPD unit, based on the trial protocol of the project, outlines the measurement approaches for the factors. For each of the factors, the SDI unit determines the safe domain/state that will be written on the T-ledger by the API located in the planning phase. In the next cycle, the SDI unit develops the next safe domains/states, considering the safety control strategies and updates the T-ledger.

**Figure 3:**
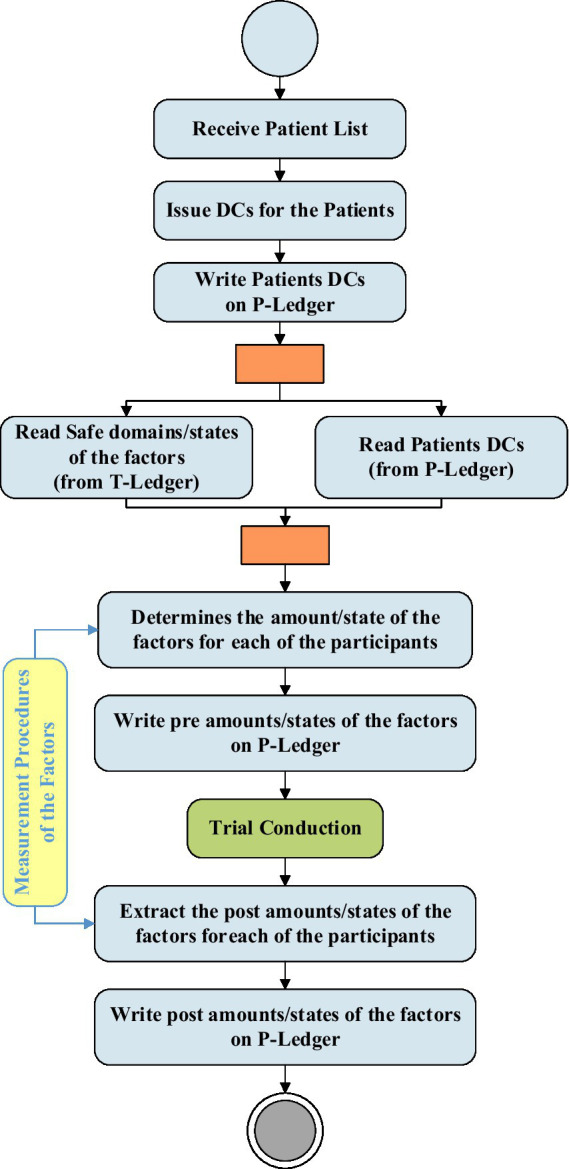
“Do” phase activities and interactions with the BCT.

Figure 3 demonstrates the activities and interactions of the do phase in the architecture. This phase, which is directly linked with the Clinical Research Organizations (CROs), is responsible for conducting the trials. As soon as a list of patients is received from a CRO, the patient DC issuer unit issues digital certificates for each of the participants and writes this information on the P-ledger. The PrT unit recalls the patients’ list from the P-ledger and safe domains/states of the trials from the T-ledger and obtains the amount/state of the factors for each of the participants using the measurement procedure provided by the MPD. After trials are conducted, the PoT unit gains the amounts/states of the factors for each of the participants and writes the post-trial results on the P-ledger.

**Figure 4:**
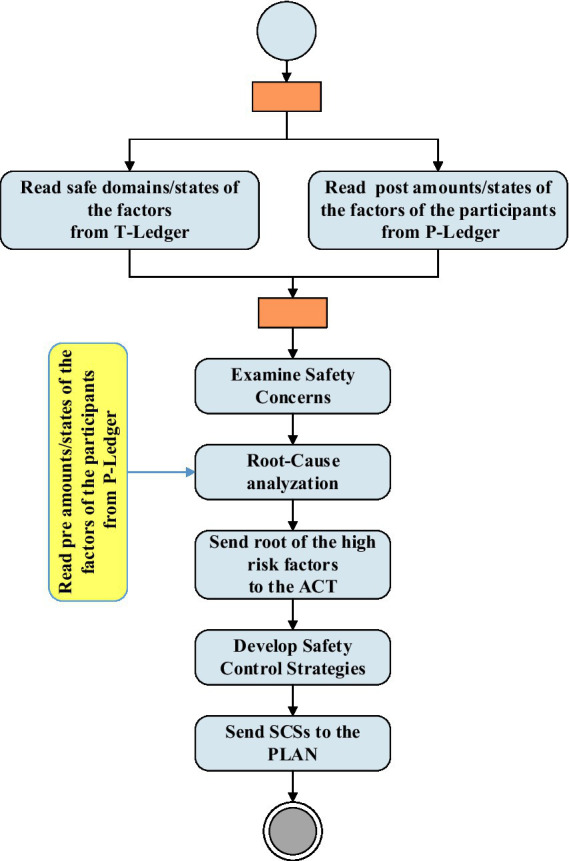
“Check” and “Act” phase activities and interactions with BCT.

Figure 4 shows the activities of the check and act phases and their interactions with BCT. As shown in the figure, the RA unit extracts safe domains/states of the factors from the T-ledger and the post amounts/states of the factors of the participants from the P-ledger and examines the safety concerns. The results of these investigations and the pre amounts/stats of the factors of the participants are used by the RCA unit to identify hazards with the potential to cause harm. This unit provides the root of the high-risk factors for the act. In the “act” phase, the roots are used by the SCSD unit to develop safety control strategies to be used in the next plan phase.

### 3.2 Blockchain structure

**Figure 5:**
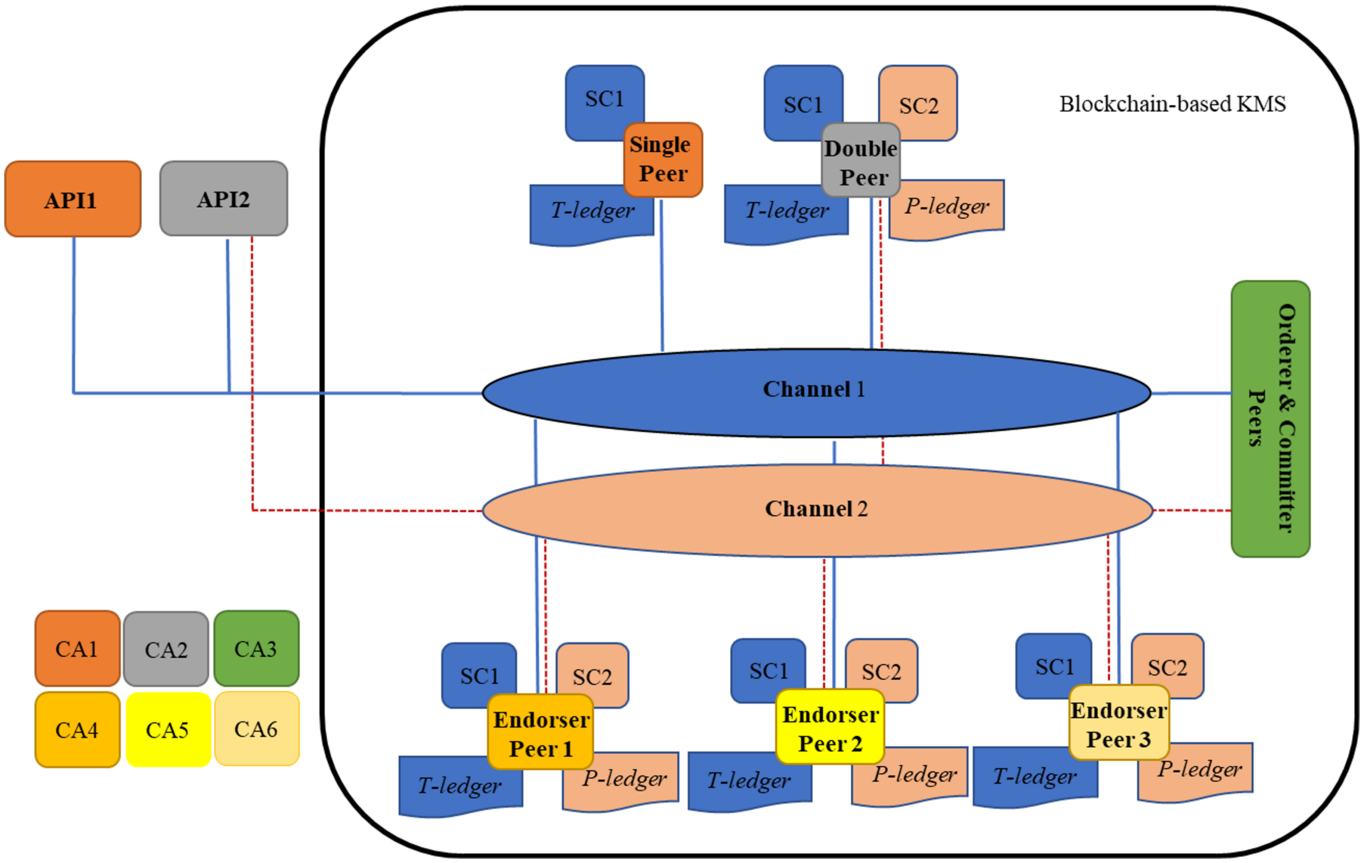
Blockchain-based KMS Structure.

The structure of the BCT is represented in Figure 5. As shown in this figure, one single, double, orderer and committer peers, and three endorser peers are represented in the architecture. A single peer holds a copy of T-ledger and its smart contract (i.e., Chain Code), and a double peer preserves a copy of T-ledger and P-ledger and their smart contracts. A single peer is capable of reading/writing information on the T-ledger through an external interrelated Application Programming Interface (API), while double peers can realize the same activities on both ledgers. API is a computing interface that is in connection with a single or double peer in the blockchain-based KMS to accomplish read and write activities from/into a ledger. In the architecture, the APIs are placed in SDI, PrT, PoT, RA, and RCA units. An endorser peer checks the details of a broadcast proposal (by a single or a double peer) and certificate details of the requester to validate the transaction. The miner peer provides ordering and committing services to the network. It packages transactions into blocks and commits/saves the transaction in the ledgers to be delivered to peers on the channel. In the BCT chain, codes are installed on the peers to perform related operations, such as instantiating, invoking, packaging, querying, and upgrading. Two channels are considered in which channel peers are sharing the same ledger and the smart contract. Channel 1 is for data exchange amongst the peers that hold T-ledger, and Channel 2 is for peers with P-ledger. In the network, six Certificate Authorities (CAs) are considered to issue a digital certificate for the peers.

### 3.3 QbD interaction with BCT

**Figure 6:**
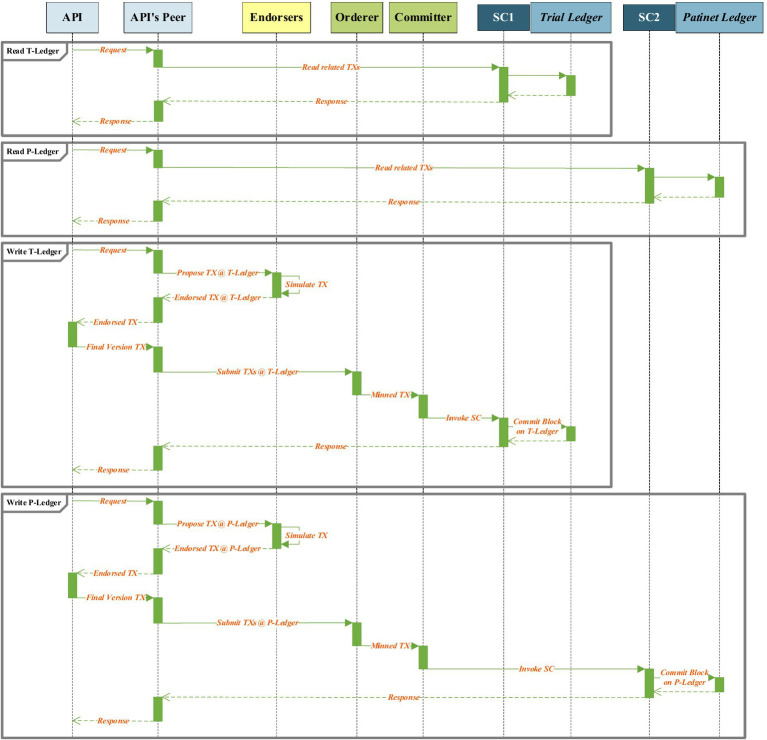
Read and write processes sequences of architecture.

In total, four kinds of interaction can be accomplished by the APIs. The API of the SDI unit demands read and write actions from the T-ledger, the API of the PrT unit requests read action from the T-ledger and write action to the P-ledger, the API of the PoT unit writes information to the P-ledger, the API of the RA unit requests read action from the P-ledger and T-ledger, and the API of the RCA unit reads information from the P-ledger. Figure 6 demonstrates the read and write procedure sequences that are requested by an API of a unit in the architecture. As shown in the figure, an instance of an API is requested to read information from a ledger. The smart contract of the API’s peer will be invoked and extract the requested information from the ledger, and forward this information to the API. To write a transaction into a ledger, the API develops a transaction proposal and forwards it to the API’s peer. This peer broadcasts the transaction proposal to the endorser peers. The endorsers simulate the transaction and provide the endorsed or refused transaction to the API through its peer for finalization. The final version of the endorsed transaction is forwarded to mining peers through the API’s peer. The mined transaction is written to the related ledger.

To operationalize the integration between QbD units and blockchain infrastructure, each functional QbD component is encoded through smart contract logic that governs how clinical data is recorded, verified, and acted upon. For example, the Critical Safety Attribute Identifier (CSAI) defines the parameters and thresholds for risk-relevant factors, which are embedded in the chaincode as configurable constants. The Risk Assessment unit RA is responsible for evaluating incoming trial data against these thresholds in real time. When a measurement is submitted, such as a participant’s lab result, it is processed as a blockchain transaction, validated using schema and threshold logic, and endorsed by authorized nodes before being committed to the ledger. The SCS component then uses these immutably stored events to determine whether automated responses, such as alerts or protocol adaptations, should be triggered. This modular control logic allows the blockchain network to support continuous QbD-driven monitoring across all PDCA phases, ensuring real-time enforcement of protocol specifications, transparent audit trails, and proactive safety governance.

## 4 Case study

To verify BCT embedded QbD, a simulation platform was developed using Java Application Descriptor (JAD). The BCN is developed based on Hyperledger Fabric technology, using open-source Hyperledger Composer. This tool is suitable for a private blockchain business environment and is based on JavaScript. The detailed fundamental implementation of BCT on a Hyperledger Fabric has been illustrated in (https://github.com/IBM/build-blockchain-insurance-app). The data from a completed pilot trial for the assessment of relative bioavailability in a generic drug product development is used for simulation.

**Figure 7:**
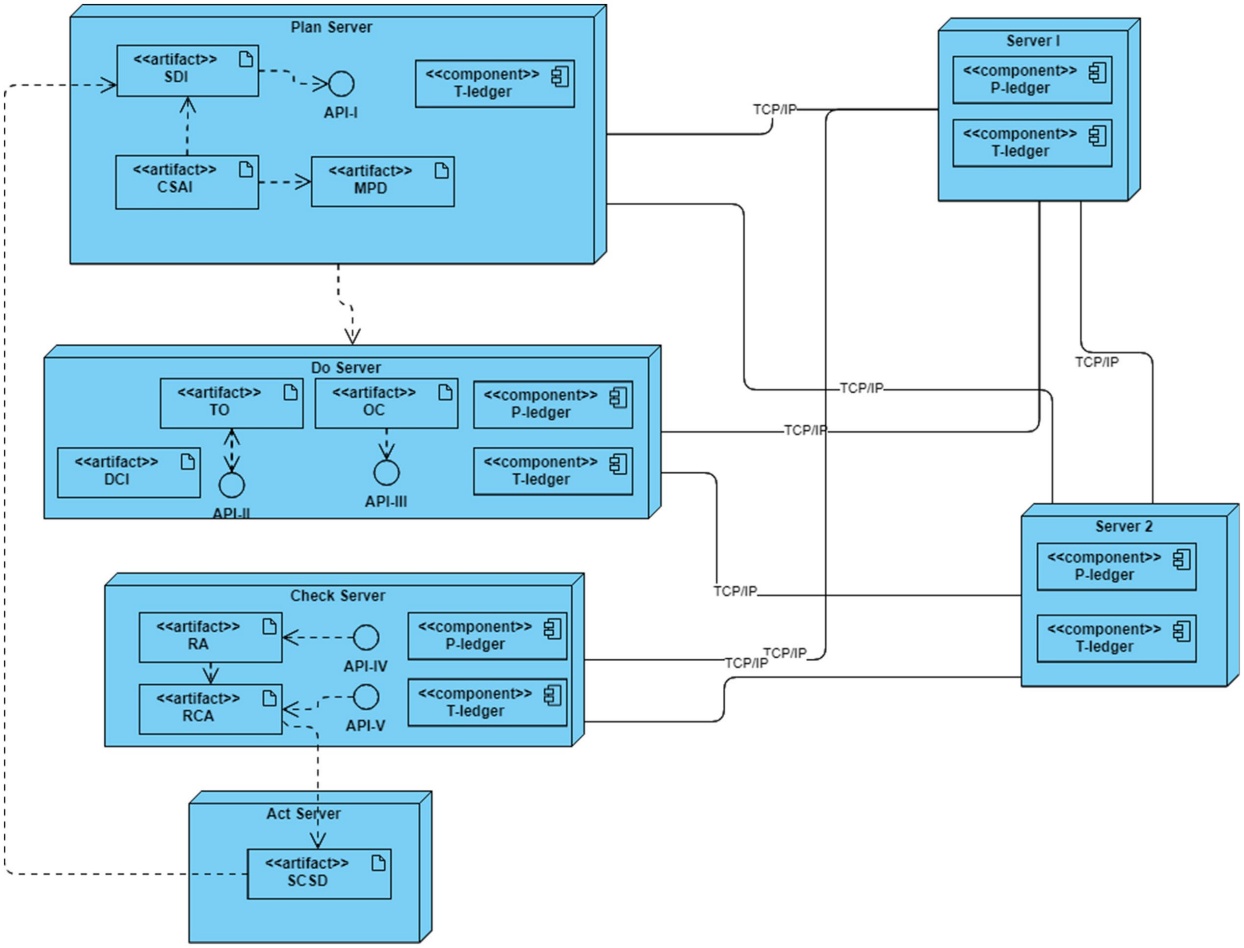
Simulation platform deployment diagram.

The deployment diagram of the developed simulation platform is represented in Figure 7. In this platform, six servers are interconnected through TCP/IP and/or Ethernet links based on the blockchain-embedded QbD architecture. Four servers simulate plan, do, check, and act entities while two more act as endorsers, and orderer/committer peers of the blockchain networks.

In the simulation platform, each of the servers may host some artifacts, components, and APIs. The artifacts exhibit the characteristics of the entities and can interact with others to obtain output results. The components of the servers execute as peers in the BCT. These peers maintain a copy of ledgers (P-ledger and/or T-ledger) and update the ledgers when an alteration happens in the network. There are three roles for a component—endorser, committer, and orderer. All the components have been designed such that a component is always a committer. The components of server 1 and server 2 are designed to perform a role in the endorsing and ordering of transactions. API is an intermediary software with a set of definitions and protocols that allows artifacts to talk with components. Plan, do, and check server host APIs to interact with the blockchain network.

**Figure 8:**
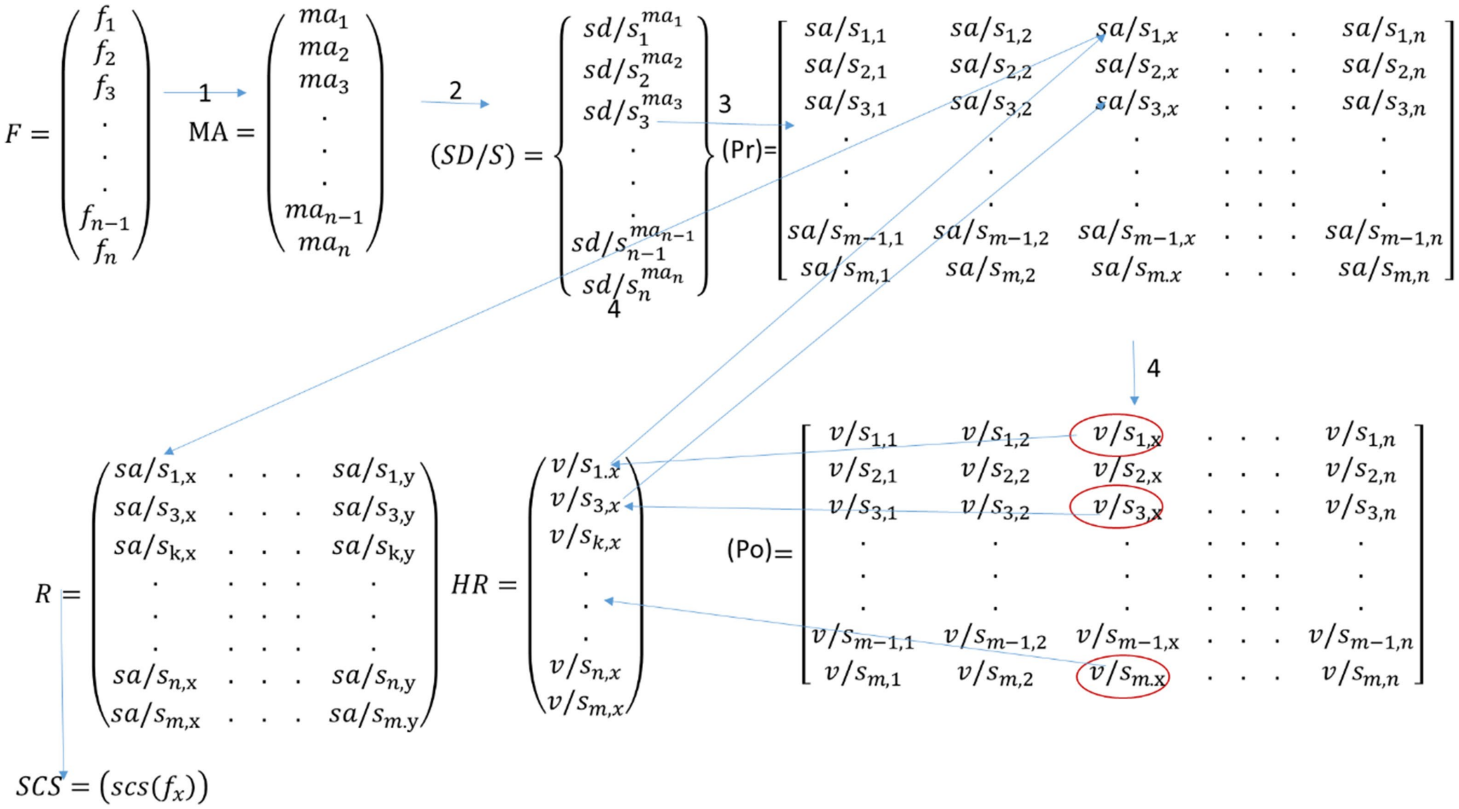
Matrix notation of the QbD interactions in the architecture.

Figure 8 represents the illustration of the interactions in the architecture. Matrix notation is used to simplify the presentation of interactions that are performed in the simulation platform. Three different types of matrices are used in this illustration. Round brackets are for representing information that will be stored on local data repository systems. The information in the square brackets will be stored on P-ledger, and the curly brackets are used to represent the information that will be stored on T-ledger in the blockchain. As shown in the figure, each artifact in the platform generates matrix(s) that are used as input for the interrelated artifacts and/or components.

Using feedback from experts, the artifacts of the plan server extract the factors of the trial, their measurement procedures, as well as the safe domains/states. As shown in the Table 1, four subjective factors (F1–F4) and one objective factor (F5) are selected by the CSAI as the elements that affect the critical safety attributes of the trial, and the expected measurement approaches for each of the factors are provided by the MPD and the safe domains/states of the factors are identified by the SDI all according to the data available on the protocol. The MPD unit stores the extracted measurement approaches on the local database, and this information is shared with the do server. It is SDI’s responsibility to develop a proposal to write the extracted safe domains/states of the factors to the T-ledger through the API-1. As soon as the developed proposal is endorsed, the transactions are written to the ledger.

**Table 1:** Critical safety factors, their measurement methods, and safe domains/states.

Factor	Measurement approach	Safe domain/state
F1: Blood pressure (BP)	Every 30 min/24 h Breakfast: 8:00–80:30 Lunch: 12:00–13:00 Dinner: 18:00–18:40	80–130 mmHg
F2: Heart rate (HR)	Every 15 min/24 h Male: walking-Max 60 min/24 h Female: walking-Max 50 min /24 h Watch TV: Max 30 min (No action) Use Phone: Max 30 min	Age <40: 90–153 b/min Age 41–45: 88–149 b/min Age 46–50: 80–145 b/min Age 51–55: 83–140 b/min Age: 56–60: 80–136 b/min Age: 61–65: 78–132 b/min
F3: Blood Sugar (BS)	F3.1: Fasting F3.2: 30 min before meal F3.3: 60 min after meal F3.4: Bedtime Breakfast < 1,100 calorie Lunch< 1,200 calorie Dinner < 800 calorie	Fasting: less than 100 mg/dL Before meal: 70–130 mg/dL After meal: less than 180 mg/dL Bedtime: 100–140 mg/dL
F4: Stay in bed	Before administration After administration Sleep style: Stargazer or Skydiver	Before administration: 60 min After administration: 240 min
F5: Ambient Temperature	Every 30 min/24 h	19–25 °C

The do server started to work when the measurement approaches are provided by the plan, and a list of the eligible participants is provided to the DCI unit. The DCI stores all the personal information of the participants on the local database and reads the required factors from the T-ledger, and develops a transaction for each of the participants. This transaction contains important demographic information of the participants, the value of the factors (considered as N/A), and the status of the conduction that is marked as “Initial.” The developed transactions are written to the P-ledger by the DCI unit. The artifact of the TO reads the transactions with “Initial” status from the P-ledger, the safe domains/states of the factors from the T-ledger, and the measurement approach of the factors from the local database to develop an order that enforces the CRO for conducting the trial according to the provided instructions. For each of the participants, the OC collects the outcomes of the factors before and after drug administration and develops transactions to be written in the P-ledger that holds the status of “Before” or “After.” Table 2 provides the information extracted from the P-ledger of the pilot trial.

**Table 2:** Extracted data from the P-ledger of the pilot trial.

Participants	Demographic information			Before administration								After administration							
	Sex	Age	Weight (Kg)	F1	F2	F3.1	F3.2	F3.3	F3.4	F4	F5	F1	F2	F3.1	F3.2	F3.3	F3.4	F4	F5
P1	M	55	70	129	122	90	88	167	103	57	23	117	101	90	129	163	103	231	22
P2	F	48	60	126	89	77	117	153	110	56	23	103	137	77	125	146	110	227	22
P3	M	39	72	86	140	93	127	144	102	58	23	94	152	93	77	172	102	234	22
P4	F	52	66	126	82	99	88	146	111	56	23	96	101	99	126	142	111	227	22
P5	M	48	80	103	126	85	79	133	107	56	23	83	89	85	97	166	107	227	22
P6	F	35	76	86	130	98	106	135	104	60	23	85	122	98	78	167	104	240	22
P7	F	25	58	98	105	82	114	155	121	52	23	105	133	82	91	155	121	215	22
P8	M	36	74	121	152	90	128	134	135	51	23	84	185	90	119	179	135	214	22
P9	M	28	78	103	152	98	107	166	130	58	23	116	124	98	98	179	130	234	22
P10	F	56	60	111	125	96	119	142	110	57	23	92	140	96	107	165	110	230	22
P11	F	47	65	103	82	87	117	155	135	55	23	105	136	87	97	142	135	225	22
P12	M	66	75	121	143	78	85	159	126	53	23	109	97	78	119	175	126	219	22
P13	M	26	68	97	92	97	108	163	107	57	23	113	126	97	91	147	107	232	22
P14	F	40	76	122	101	95	123	131	116	54	23	81	144	95	120	152	116	222	22
P15	M	49	79	81	109	92	83	176	136	54	23	126	94	92	71	157	136	223	22
P16	M	56	83	86	137	78	103	131	137	54	23	81	119	78	77	172	137	221	22
P17	F	60	75	88	140	92	72	177	139	53	23	127	80	92	80	173	139	218	22
P18	F	31	70	87	119	79	84	134	116	55	23	84	95	79	78	162	116	226	22
P19	M	42	83	86	118	75	92	154	101	52	23	104	106	75	77	161	101	217	22
P20	M	49	85	112	149	94	79	148	103	59	23	98	89	94	108	178	103	237	22
P21	F	35	71	104	117	83	106	144	127	57	23	94	123	83	99	161	127	230	22
P22	M	59	79	94	134	90	109	134	124	53	23	84	127	90	86	170	124	220	22
P23	F	61	67	82	78*	99*	83	142	134	59	23	92	152*	98	73	140	134	238	22
P24	F	50	79	109	99	86	108	161	134	51	23	111	126	86	105	151	134	212	22
P25	M	44	81	109	121	78	129	164	112	59	23	114	151	78	105	163	112	236	22
P26	F	39	66	104	122	86	113	179	130	53	23	129	132	86	99	163	130	219	22
P27	M	36	77	124	132	81	113	175	137	56	23	125	131	81	123	169	137	228	22
P28	F	64	72	97	80*	98*	95	143	102	53	23	93	146*	97	90	143	102	219	22
P29	M	24	72	89	105	84	104	176	114	56	23	126	121	84	81	154	114	227	22
P30	M	29	80	109	132	81	105	155	134	60	23	105	121	81	105	169	134	239	22

The underline and * values indicate block chain-derived data suggests the patient may be at medium risk.

In the check server, the RA unit reads the safe domains/stats from the T-ledger and recalls the after-drug administration transactions from the P-Ledger and highlights the participants that contain unsafe values. As shown in the table, after drug administration, F2 values of the P3, P8, P23, and P28 are on the unsafe borderline. RA forwards the ID of the participants to the RCA unit for further consideration. The RCA reads all the transactions related to the selected participants from the P-ledger and their safe domains/states from the T-ledger to perform a root cause analysis for finding relations among the demographic information of the patricians, the value of the factors before administrations, and the unsafe values. Based on the P23 and P28 information, it is determined that the female participants’ age between 60 and 65, low heart rate, and high fasting blood sugar may reveal an unsafe value of high heart rate after drug administration. It also committed that the male participants in the 35–40 age range (i.e., P3 and P8), with high heart rate, and high before-meal blood sugar level, may show the unsafe value of heart rate after drug administration. These roots of the unsafe values are forwarded to the SCSD unit in the act server, where the unit develops a safety control strategy as a recommendation for the plan server. One possible safety control strategy is to exclude or limit the future participants holding the same specifications. This recommendation will be used by the artifacts in the plan in the next trials.

## 5 Discussion

The experience gained during the case study implementation allows us to draw some conclusions about the operation of the BCT-embedded QbD. It was verified that the system works as specified in both the QbD and the BCT sides, thus validating the robustness of the developed system. Additionally, the artifacts, components, and APIs of the developed system were proven by their accurate reactions to the assigned responsibilities. The system identifies some unsafe borderline values, which are neglected during the real-life pilot trial. This system provides trustworthy data integration with clinical trial management and clinical data management systems, with features to extract sponsors, ethics committee, and regulators features to construct a knowledge management system to be used by the CROs, based on participant safety as the main enabler of the quality in clinical research. However, some of the following questions remain for investigation:

1. How to realize the real development and application of the developed system?
2. How to persuade the sponsors, CROs, and regulating authorities to use current technologies in this field?
3. How do we ensure security during the process of collaboration?

To realize the gap between clinical research, advanced technologies, and the proposed architecture, it is mandatory to negotiate between all stakeholders in a drug life cycle to discuss how to implement this architecture in clinical trial studies to grasp its advantages and disadvantages. Because of the absence of clear standards of regulating authorities or little standardization, the stakeholder may have challenges. By growing the BCT and related infrastructures, the standards should be upgraded. Moreover, the quality concerns in the clinical trials may improve drastically with these approaches. In addition, the potential security risks of BCT can be mitigated by cybersecurity professionals as safely as possible.

BCT-enabled QbD enhances clinical trial quality by providing a rigorous, data-driven approach to managing participant safety and trial data integrity. Central to this approach is the redefinition of core QbD elements Quality Targets (QTs), Quality Attributes (QAs), Critical Safety Attributes (CSAs), and Safety Control Strategies (SCSs), which systematically prioritize the protection of participants and the reliability of trial outcomes. By leveraging BCT’s immutability, the system ensures that critical safety parameters and measurement protocols are transparently recorded and securely maintained. This immutability addresses a major regulatory and scientific concern: the prevention of data manipulation and loss, thereby guaranteeing the accuracy and credibility of clinical trial data essential for regulatory review and scientific interpretation.

The integration of BCT with the iterative QbD phases facilitates continuous risk assessment and quality improvement, ensuring compliance with GCP. During the Plan phase, the Critical Safety Attribute Identifier, Measurement Procedure Determiner, and Safe Domain Identifier collaboratively identify safety-critical factors, define their measurement methodologies, and establish safe operational domains, with all decisions recorded on the T-ledger. The Do phase operationalizes these plans by issuing secure digital certificates to trial participants via the P-ledger, verifying identity while logging pre-trial and post-trial factor states using validated measurement procedures. In the Check phase, comprehensive risk assessment and root cause analysis units analyze the data stored on both ledgers to identify deviations and safety risks. The Act phase then uses these insights to develop and update Safety Control Strategies, ensuring that subsequent trial cycles proactively mitigate identified risks. This closed-loop process enhances participant safety by embedding proactive, data-backed decision-making into trial conduct, exceeding traditional audit-based quality assurance methods.

Moreover, the BCT-enabled QbD strengthens collaboration, accountability, and compliance across all clinical trial stakeholders, including sponsors, CROs, regulators, and independent auditors. The system supports multiple peer roles: endorser, committer, and orderer that manage transaction validation, block creation, and ledger updates, ensuring robust governance and data provenance. The separation of patient-specific data (P-ledger) and trial protocol data (T-ledger) provides compartmentalized data control aligned with privacy regulations while enabling comprehensive traceability. These design choices promote regulatory compliance with data integrity standards such as the FDA’s 21 CFR Part 11 and the EMA’s guidelines on computerized systems. The approach directly addresses key challenges in clinical trials, participant safety, data accuracy, and transparency by integrating advanced technology with systematic quality management, thus aligning with and potentially exceeding evolving expectations in clinical trial oversight.

The BCT-enabled QbD distinguishes itself through its structured, iterative approach to quality improvement and participant safety management in clinical trials. By integrating core QbD elements and embedding these within permissioned ledgers using Hyperledger Fabric, the system ensures rigorous data integrity, traceability, and dynamic risk control. This contrasts with more generalized blockchain applications in clinical research that primarily focus on securing data or improving transparency without the systematic embedding of quality frameworks. For instance, Leiva et al. (2025) and Castro et al. (2024) emphasize BCT’s role in enhancing data governance and transparency, particularly when integrated with AI, but their work does not specifically operationalize quality principles in trial management. Instead, their platform targets broad data integrity and recruitment optimization, especially in resource-limited settings, highlighting scalability and regulatory compliance challenges not fully addressed in simpler BCT implementations (40, 43).

Other platforms, as mentioned in Moatari-Kazerouni et al. (2024) and Nnadiekwe et al. (2024), focus extensively on enhancing stakeholder interoperability and streamlining communication through real-time data sharing enabled by smart contracts (44, 45). Their systems emphasize consent management, adverse event reporting, and participant engagement using Ethereum-based networks or permissioned blockchains with smart contract automation. While these platforms improve operational efficiencies and data security, they typically lack the explicit incorporation of iterative quality improvement cycles or safety domain-specific analytics that are central to the QbD. Additionally, Cihan et al. (2025) present an implementation of BCT with FAIR data principles for epidemiological reporting, demonstrating improved data findability and interoperability, but the platform is oriented more towards public health surveillance rather than tightly controlled clinical trial safety management (46).

### 5.1 How blockchain enhances the implementation of QbD in clinical trials

BCT reinforces the stakeholder in clinical trials by offering a secure, transparent, and immutable infrastructure for managing critical trial data, processes, and safety controls. At its core, QbD emphasizes proactive risk management, data-driven decision-making, and built-in quality from the earliest stages of clinical trial planning. BCT strengthens these aims in several specific ways:

- Immutable and time-stamped records for risk-based quality assurance; Each QbD activity, such as defining Critical Quality Attributes, identifying risks, or updating trial protocols, can be logged on a blockchain ledger, ensuring traceable, tamper-proof documentation. This is particularly important in demonstrating regulatory compliance and maintaining audit trails. For example, updates to safety protocols based on interim analysis are stored immutably, offering clear evidence of compliance with GCP standards.
- Decentralized trust and multi-stakeholder collaboration in clinical trials involve diverse actors, sponsors, CROs, investigators, and regulators, each with different data ownership and governance interests. The framework ensures all stakeholders have access to synchronized, validated information while preserving role-based permissions. This supports QbD’s call for cross-functional, multidisciplinary input during risk assessments and control strategy formulation.
- Real-time monitoring and feedback; smart contracts automate conditional logic and real-time alerts. For example, if a predefined adverse event threshold is crossed in a specific patient population, a smart contract can automatically trigger a safety review workflow. This aligns with QbD’s principle of continuous process monitoring and adjustment, enhancing patient safety and operational agility.
- Dual-ledger architecture for data stratification; the separation of P-ledger (protocol, risk assessment, operational metadata) and T-ledger (transactional, patient-level data) ensures secure stratification of information. This allows QbD planning and evaluation activities to be conducted without compromising patient confidentiality critical under regulations like GDPR.

A concrete justification for the use of BCT-enabled QbD is in adaptive trials, such as those in oncology, where design flexibility is essential for ethical and scientific reasons. During the Plan phase, adaptive rules are encoded as smart contracts on the blockchain. In the Do phase, patient outcomes feed into these rules, enabling real-time decision-making based on pre-validated criteria. BCT ensures these adaptations are logged immutably, with precise timestamps and cryptographic proof of authenticity. During Check, oversight bodies can independently audit these changes, verifying both compliance and rationale. If adverse safety patterns emerge in a subgroup, the Act phase initiates targeted protocol amendments, which are again logged for transparency and future audits.

In decentralized clinical trials, where trial operations span across varied geographies and infrastructure, BCT-enabled QbD supports quality assurance through localized planning and centralized evaluation. For example, in the Plan stage, site-specific risk mitigation strategies—like tailored e-consent workflows or sensor calibration protocols—are defined and stored on the blockchain ledger. As the trial progresses (Do), data from wearable devices and remote diagnostics are logged across distributed nodes. Smart contracts can automatically trigger alerts when anomalies or deviations are detected, activating the Check process, which validates trends and identifies root causes. In the Act phase, remote monitoring practices or data collection protocols can be refined to prevent recurrence, with all changes being traceably versioned.

### 5.2 Security considerations: potential attack surfaces and mitigation strategies

Despite the robustness of permissioned blockchain infrastructures like Hyperledger Fabric, the proposed system must address several potential attack surfaces. Insider threats, such as unauthorized ledger access or policy tampering by misconfigured peer roles, are mitigated using role-based access control (RBAC) and certificate authority–based identity verification. Data injection or manipulation attacks are prevented via smart contract validation logic and endorsement policies that require transaction approval by multiple trusted entities (e.g., sponsor, CRO). To counter privacy risks, particularly regarding patient-specific data, the system uses hashed references and private data collections. Denial-of-service attacks targeting peer nodes are handled through resource quota enforcement and peer throttling. Additionally, ledger tampering is rendered computationally infeasible by the immutable, cryptographically chained blocks and auditable logs. Periodic ledger audits, chaincode verification, and zero-trust network configurations provide layered defense, ensuring that both data integrity and operational continuity are maintained across the clinical trial lifecycle.

To address participant privacy and align with GDPR and HIPAA requirements, particularly the right to withdraw consent and the right to be forgotten, the proposed system employs an off-chain reference model. Sensitive personal data are stored securely off-chain in encrypted databases, while only hashed pointers and metadata are recorded on-chain. This approach enables consent revocation by allowing off-chain data to be deleted or access-restricted without altering the blockchain’s immutable record. Additionally, pseudonymization techniques are used to decouple identifiable information from clinical data, ensuring that patient identities remain protected even if on-chain references are accessed. These combined strategies offer a compliant and flexible privacy-preserving framework within a decentralized clinical trial environment.

### 5.3 Stakeholder engagement and adoption strategy

Successful adoption of the proposed BCT-enabled QbD framework requires a structured, multi-phase stakeholder engagement strategy to ensure operational alignment and real-world feasibility. Key stakeholder groups, including sponsors, CROs, regulatory authorities, and site investigators, must be engaged through targeted efforts focused on awareness, capacity building, and collaborative governance. Initial engagement should emphasize the system’s value proposition, particularly its potential to enhance patient safety, ensure data integrity, and streamline regulatory compliance. To support adoption among non-technical users, hands-on training modules and simulation environments can facilitate digital literacy and reduce implementation resistance.

Furthermore, early stakeholder involvement during protocol design allows for the incorporation of domain-specific insights into the system’s risk definitions and control strategies, ensuring alignment with evolving regulatory standards such as ICH E6(R3) and M11. Clear assignment of blockchain roles, such as endorsers, committers, and auditors, should be formalized through a governance framework that delineates responsibilities and access control. To build credibility and refine deployment strategies, pilot-phase collaborations with innovation-oriented institutions are recommended. Finally, continuous feedback mechanisms via steering committees and stakeholder review boards are essential for iterative improvement, regulatory responsiveness, and sustained trust across the clinical trial ecosystem.

### 5.4 Study limitations

While the BCT-enabled QbD offers a transformative approach to operationalizing PDCA in CTs, several limitations warrant critical consideration. First, scalability remains a significant challenge, especially when dealing with high-frequency data inputs such as those from wearable sensors or real-time remote monitoring devices in decentralized clinical trials. Second, regulatory alignment poses ongoing difficulties, particularly with global privacy laws such as the GDPR and HIPAA. BCT’s inherent immutability can conflict with patients’ legal rights to data erasure or correction, presenting unresolved tensions in clinical contexts where ethical and legal compliance is non-negotiable. Techniques like off-chain storage, zero-knowledge proofs, or selective disclosure might be applicable, but their deployment remains technically intricate and under-regulated, limiting their immediate applicability in multi-jurisdictional trials.

Third, interoperability across stakeholders and digital systems is limited, often requiring custom APIs, middleware solutions, or manual reconciliation with legacy electronic data capture, clinical trial management systems, or laboratory information management systems. This adds operational overhead, potentially undermining the efficiency gains expected from automation and smart contract execution. Moreover, the steep learning curve and technological unfamiliarity among clinical personnel can hinder adoption, requiring comprehensive training, change management strategies, and institutional investment in digital literacy.

Finally, the lack of large-scale empirical validation across therapeutic areas and trial phases restricts the generalizability of the framework implementations. As noted in studies by Castro et al. (2024) and Cihan et al. (2025), real-world deployments are still limited and often lack standardized benchmarks for performance evaluation (43, 45). This calls for robust, longitudinal studies and multi-site collaborations to fully substantiate the framework’s reliability, regulatory robustness, and cost-effectiveness in diverse clinical environments.

The sample size of the study for proof of concept was based on a pilot clinical trial with 30 participants. This sample size is insufficient for obtaining precise and comprehensive results. By employing the data of different clinical trials with a larger population in three different phases of trials can be generalized the feasibility and practicability of the system.

To scale the framework from a Phase I bioavailability study to later-phase clinical trials, the system must adapt to increased complexity, particularly in ensuring patient safety, protocol adherence, and regulatory compliance across diverse clinical settings. In later-phase trials, the inclusion of patient populations introduces greater variability and risk, necessitating robust mechanisms for real-time monitoring and auditability. BCT can be extended through smart contracts to automate the detection and reporting of adverse events, enforce protocol compliance dynamically, and ensure immutable logging of trial activities. These capabilities enhance transparency and trust among stakeholders, including investigators, sponsors, and regulators.

To address the data volume and integration challenges typical of multicenter Phase II–IV trials, the framework should be capable of supporting interoperability with EHR systems and allow for decentralized yet secure data sharing across trial sites. Patient-centric features such as dynamic consent management and secure, encrypted data access further ensure ethical engagement and compliance with privacy regulations. The integration of AI can further augment the system by enabling predictive analytics for risk-based monitoring, early detection of protocol deviations, and optimization of trial operations. For scalability, off-chain storage solutions can be employed to maintain efficiency without compromising data integrity or security.

Lack of previous extensive research studies: currently, scant previous research and inadequately developed models fail to answer how and where QbD is effectively applicable and how these approaches can be employed in clinical trials efficiently. Further researches are needed to overcome this challenge. In future works, the architecture can be used in real-life clinical trials, and the medical cyber-physical system can be used for real-time monitoring of the patients and equipment. Artificial intelligence-based tools can be used to estimate the efficacy and accuracy of the architecture.

As this study presents a conceptual and exploratory framework, stress testing and performance benchmarking (e.g., latency and throughput) were not conducted due to infrastructure limitations; however, we acknowledge this as a limitation and propose detailed scalability evaluation as part of future system validation. Given the prototype stage of development, isolated component analysis (ablation study) was not conducted; however, we recognize this as a limitation and identify it as an important area for future empirical validation to assess the individual contributions of each QbD module. The case study is illustrative and not statistically powered, so we recommend conducting performance benchmarking in future real-world pilot implementations. For future studies, we recommend conducting analysis using real-time datasets, which were not possible in the current study.

## 6 Conclusion

In this study, a blockchain-embedded quality by design architecture for the clinical trials stage of drug development is developed, and the applicability of the architecture is tested using the data of a trial with 30 participants. The unified modelling language is used to present the architecture. The architecture contains two main parts, including QbD and BCT, that are integrated through the internet. The plan, do, check, and act framework is used to develop the QbD, while blockchain is employed to build a distributed, immutable ledger technology for storing the safety-related knowledge of the project. The architecture is working in a way that, first, the protocol of a trial is screened to highlight the parameters that might raise safety concerns for the participants, then the value of these subjective and objective concerns before and after drug administration is extracted, and finally, using root cause analysis, a control strategy is developed for the next trials. All the safety-related data of the participants is stored on a two-blockchain ledger. The reasons to employ blockchain instead of conventional information repository techniques are: the stored information cannot be altered or deleted, a high level of data security, including less risk of duplicate entry or fraud, time-stamped, transparency, scalability, and traceability. Future implementation of blockchain-based QbD frameworks requires not only technical validation but also strategic alignment with key industry stakeholders, including sponsors, regulators, and research organizations. Rather than questioning how to persuade sponsors to adopt such systems, efforts should focus on demonstrating measurable benefits such as enhanced data integrity, reduced operational risks, and streamlined regulatory compliance. Integration with emerging regulatory frameworks, such as the FDA and EMA blockchain pilot initiatives, can provide a structured pathway for validation and acceptance. Additionally, moving beyond small-scale proofs of concept toward scalable deployment across multi-center clinical trials will be essential to demonstrate robustness and interoperability. At the same time, cybersecurity and data privacy must remain at the forefront, ensuring that distributed data storage and smart contract automation do not compromise sensitive clinical information. Overall, the next phase of research should emphasize regulatory integration, scalability, and security as key enablers for widespread adoption of blockchain-enabled QbD systems in clinical development.

## Data Availability

The raw data supporting the conclusions of this article will be made available by the authors, without undue reservation.
